# Quality evaluation of mycelial *Antrodia camphorata *using high-performance liquid chromatography (HPLC) coupled with diode array detector and mass spectrometry (DAD-MS)

**DOI:** 10.1186/1749-8546-5-4

**Published:** 2010-01-29

**Authors:** Sandy Shuo Zhao, Kelvin Sze-Yin Leung

**Affiliations:** 1 Department of Chemistry, Hong Kong Baptist University, Kowloon, Hong Kong SAR, China

## Abstract

**Background:**

*Antrodia camphorata *(AC) is an important fungus native to Taiwanese forested regions. Scientific studies have demonstrated that extracts of AC possess a variety of pharmacological functions. This study aims to identify the full profile fingerprint of nucleosides and nucleobases in mycelial AC and to assess the quality of two commercial mycelial AC products.

**Methods:**

High-performance liquid chromatography coupled with diode array detector and mass spectrometry was employed to identify the major components in mycelial AC. The chemical separation was carried out using a gradient program on a reverse phase Alltima C_18 _AQ analytical column (250 × 4.6 mm, 5 μm) with the mobile phase consisting of deionized water and methanol.

**Results:**

Ten nucleosides and nucleobases, two maleimide derivatives, and a sterol were identified as the major constituents in mycelial AC. These groups of chemical compounds constitute the first chromatographic fingerprint as an index for quality assessment of this medicinal fungus.

**Conclusions:**

This study provides the first chromatographic fingerprint to assess the quality of mycelial AC.

## Background

*Antrodia camphorata *(M. Zang & C.H. Su) Sheng H. Wu, Ryvarden & T.T. Chang (Polyporaceae) is a parasitic fungus on decayed wood or the inner wall of the heartwood of *Cinnamomum kanehirai *hay, a tree endemic to Taiwan. Before *Antrodia camphorata *(AC) was first officially classified as a species in 1990, its medicinal value had been greatly appreciated for many decades. This highly valuable fungus is widely recommended by the traditional Chinese medicine practitioners for food intoxication, vomiting, and poisoning [1]. In addition, it was shown effective to improve liver and stomach immunity [2]. Due to its medicinal value and scarcity in nature, excessive forestry cutting down of *Cinnamomum kanehirai *is prohibited by the Taiwanese government [3].

After the success in mass production of AC by artificial cultivation, a series of health supplements formulated from AC has been launched with high market value [3], and are increasingly popular in the Taiwan, Japan, and other Asian regions. Counterfeit over-the-counter AC products have been found and reported. However, there is no reliable quality assessment method to evaluate the AC-based health supplements.

Currently, information regarding the bioactivity, pharmacology and, in particular, the chemical composition of AC is scarce [3-5]. Most AC research has been focused on the crude isolated fractions, which are subjected to pharmacological screening or therapeutically evaluation [6-12]. Recent research into the bioactivity of AC, in treating liver diseases [13] with its biochemical mechanisms derived.

Triterpenoids and polysaccharides have been the focus of numerous AC studies due to their well-known pharmacological activities [7,12,14]. In mycelial AC, these bioactive chemicals include amino acids [14,15]; lipopolysaccharides [16]; nucleosides and nucleobases such as adenosine, cordycepin, cytidine, and thymine [10,11,17,18]; maleic acid and succinic acid derivatives [6,19,20]; benzenoids [21]; phenol and tocopherols [8,22]; 5'-nucleotides [14]; and diterpenes [23].

No chemical standardization or quality evaluation methods have been established for AC. As widely used in the quality control practices for other herbs, chromatographic fingerprinting is simple and useful. Thus, this study aims to identify the full profile fingerprint of nucleosides and nucleobases in mycelial AC by using high-performance liquid chromatography coupled with diode array detector and mass spectrometry (HPLC-DAD-ESI-MS) and to assess the quality of two commercial mycelial AC products.

## Methods

### Plant

Powdered mycelium and an intact fruiting body of AC were supplied by GeneFerm Biotechnology Co. Ltd of Taiwan. Samples of two over-the-counter mycelial products were purchased from a Taiwanese commercial vendor (Hung-An Pharmacy). The crude herb was morphologically and microscopically authenticated by pharmacognosist Zhongzhen Zhao at Hong Kong Baptist University. The fruiting body was cut into small pieces and ground to powder. The powder of the samples was used for analysis.

### Instrumentation

A Waters 2695 series HPLC system (Waters, USA) coupled with a Waters 2996 PDA (Waters, USA) was used. The column configuration consisted of a reverse phase C_18 _AQ column (Alltech, Alltima, 250 × 4.6 mm, 5 μm) and an Econosphere C_18 _guard column (Alltech, Alltima, 7.5 × 4.6 mm). The mobile phase consisted of deionized water (A), and methanol (B) using the gradient program as follows: 0-15 minutes, 0% B; 15-20 minutes, 0-2% B; 20-30 minutes, 2-15% B; 30-40 minutes, 15-35% B; 40-50 minutes, 35-60% B; 50-65 minutes, 60-70% B; 65-80 minutes, 70-85% B; 80-95 minutes, 85-100% B; and 95-115 minutes, 100% B. The flow rate was 1.0 ml per minute with an injection volume of 10 μl. The column was maintained at room temperature of 25°C, and the re-equilibration time of the column was maintained as five minutes before another injection. The PDA detector (Waters, USA) was set at the optimum wavelength of 260 nm.

An Agilent 1100 series HPLC-DAD system (Agilent, USA) coupled with an ion trap mass spectrometry detector was used. The system was equipped with an electrospray ionization (ESI) source and an ion trap analyzer for UV and MS data acquisition. A reverse phase C_18 _AQ (Alltech, Alltima, 250 × 4.6 mm, 5 μm) column with a 300SB-C_18 _(Zorbax, 12.5 × 4.6 mm, 5 μm) guard column was used. The signals from the mass detector were recorded and analyzed by Bruker Daltonics data analysis software (Bruker, USA). The mobile phase for the qualitative analysis of the samples consisted of 5 mM ammonium acetate in deionized water, pH 6.79 (A), and methanol (B) by using the gradient program as follows: 0-5 minutes, 0% B; 5-10 minutes, 0-2% B; 10-20 minutes, 2% B; 20-25 minutes, 2-4% B; 25-30 minutes, 4-6% B; 30-40 minutes, 6-15% B; and 40-60 minutes, 15-100% B. The flow rate was 1.0 ml per minute with an injection volume of 20 μl. The column was maintained at room temperature (25°C). The ESI-MS spectra were acquired in both positive and negative ion modes and compared on their relative sensitivities on the target compounds of interest. The capillary voltage was set at -4 kV. The full scan mass spectra were obtained from a range of m/z from 50 to 400. The nebulizer pressure was at 30 psi. The flow rate of dry gas was maintained at 6 litres per minute. Dry gas temperature was maintained at 350°C, and the collision energy was set at 2 eV.

### Solvents and chemicals

HPLC-grade solvents including methanol, acetonitrile, analytical grade chemicals including phosphoric acid, acetic acid, sodium hydroxide, and ammonium acetate, and deionized water generated from an Milli-Q water system were used for the preparation of mobile phases. Chemical standards of cytosine, cytidine, adenosine, adenine, inosine, guanine, cordycepin, uracil, and uridine (>99%; Sigma) were available for the identification of compounds in the samples.

### Sample preparation and chromatography

For the chromatographic profile of water extracts, 0.1 g of the sample was accurately weighed and extracted in 2 ml of Milli-Q water under ultrasonication for 45 minutes at room temperature. The supernatant was then filtered through a 0.45 μm Millipore filter before injecting 10 μl into the HPLC. For the chromatographic fingerprint, 0.1 g of the sample was accurately weighed and extracted in 10 ml of methanol in a conical flask under ultrasonication for 45 minutes at room temperature. The supernatant was then filtered, dried, and reconstituted into 2 ml of methanol and water (85:15). The reconstituted solution was then filtered before HPLC injection.

## Results and discussion

### Nucleosides and nucleobases as major components of water extract

The chemical components in the water-soluble fraction were characterized by comparison with authentic chemical markers and LC-ESI-MS for structural elucidation. Experimental parameters were systematically adjusted to obtain the maximum number of extractable chemical compounds for a comprehensive chemical profile. Two major chemical groups, namely polysaccharides [2,7,12,24,25] and 5'-nucleotides [14], together with nucleosides and nucleobases such as adenosine, cordycepin, cytidine, and thymine, were identified in the water extract of AC. As our previous study on *Ganoderma lucidum*, which is closely related fungus in taxonomy [3-5] and therapeutic value [26-28], also identified nucleosides and nucleobases as the major components[26], the full profile of nucleosides and nucleobases in AC can be useful in developing a fingerprint.

**Figure 1:**
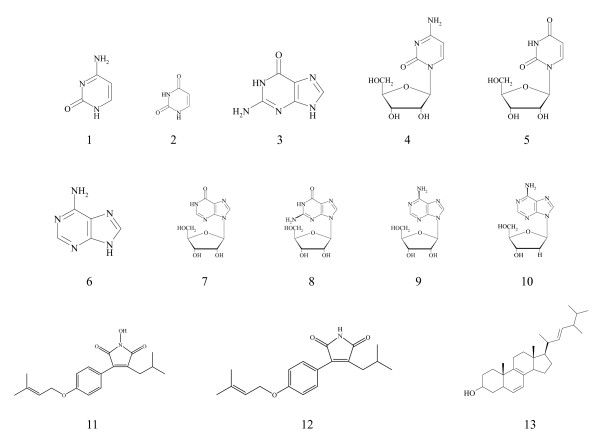
**The chemical structures of compounds in water and methanol extracts of *Antrodia camphorata***: **1**, cytosine; **2**, uracil; **3**, guanine; **4**, cytidine; **5**, uridine; **6**, adenine; **7**, inosine; **8**, guanosine; **9**, adenosine; **10**, 2'-deoxyadenosine; **11**, camphorataimide C; **12**, 3-isobutyl-4-[4-(3-methyl-2-butenyloxy)phenyl]-1H-pyrrole-2,5-dione; **13**, ergosterol.

**Figure 2:**
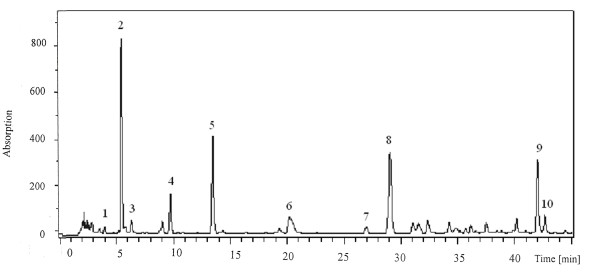
**The HPLC-DAD chemical profile of the water extract of mycelial *Antrodia camphorata***: **1**, cytosine; **2**, uracil; **3**, guanine; **4**, cytidine; **5**, uridine; **6**, adenine; **7**, inosine; **8**, guanosine; **9**, adenosine; **10**, 2'-deoxyadenosine.

An extensive determination of the nucleoside and nucleobase profiles in the water extract of AC was therefore conducted. Ten nucleosides or nucleobases (namely, cytidine, cytosine, adenine, adenosine, uridine, uracil, guanine, inosine, guanosine, and 2'-deoxyadenosine) were identified in the mycelia AC (Figure 1). Based on the ESI-MS, the molecular and product ions were observed in the forms of [M+H]^+^, [M+K]^+^, and [M+Na]^+^. Positive scan mode was chosen because of nucleosides and nucleobases are basic compounds and are more likely to be ionized with cations such as H^+^, K^+^, and Na^+^, thus facilitating the ESI-MS detection. Figure 2 shows the chromatographic profile of the water extract of mycelial AC.

### Comprehensive chemical profile of AC

The appropriate solvent should be used to extract as many groups of representative chemical classes and compounds as possible to depict the chemical profile of a medicinal material. Methanol and n-hexane were employed for extracting compounds from mycelial and fruiting body AC [18,21]. In the present study, five solvents of different polarities (water, methanol, ethanol, chloroform, and n-hexane) were evaluated with regard to their extraction efficiency. We found that methanol was able to extract most chemical compounds. This solvent was chosen to maximize the number of compounds extracted from our AC samples.

### HPLC-DAD chromatographic fingerprint

To ensure proper elution and separation of all characteristic compounds, polarities and pH of mobile phases were tested. The organic component of the mobile phase was alternated between methanol and acetonitrile. As the present 5 mM ammonium acetate and methanol offer a basic aqueous environment for the analytes, an acidic counterpart of aqueous mobile phase with 0.1% phosphoric acid in deionized water, pH 2.19 and methanol was tested. In addition, a neutral aqueous mobile phase of deionized water and methanol was also tested. The use of neutral aqueous mobile phase showed more peaks but at the expense of peak shape and symmetry. Methanol is the best choice of organic components to facilitate elution of ergosterol, which is only compatible with solvents of lower polarity.

### Method validation

To verify column performance and appropriateness of the chromatographic conditions, the number of theoretical plates, selectivity, resolution and peak symmetry values were determined as the indicators of separation efficiency. Resolution values were all higher than 1.5, which indicates good separation. Six replicate injections of a sample solution were performed to assess the precision of the methanol. The relative standard deviation (RSD) of relative retention time and relative peak area were less than 0.64% and 4.07%, respectively. Another six independently prepared samples were assessed for the repeatability of the method. The RSD of relative retention time and relative peak area were 0.77% and 6.89%, respectively. The sample stability was determined by three repetitive injections of a sample solution after three days of storage at room temperature. The RSD of relative retention time and relative peak area were 0.67% and 7.45%, respectively.

### Qualitative chromatographic fingerprint

**Figure 3:**
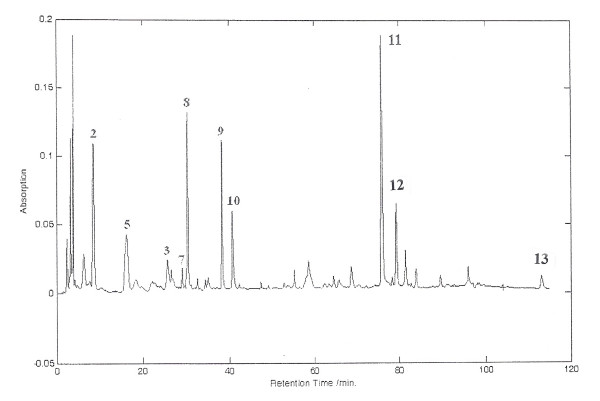
**The established HPLC-DAD fingerprint of methanol extract of mycelial *Antrodia camphorata***: **2**, uracil; **3**, guanine; **5**, uridine; **7**, inosine; **8**, guanosine; **9**, adenosine; **10**, 2'-deoxyadenosine; **11**, camphorataimide C; **12**, 3-isobutyl-4-[4-(3-methyl-2-butenyloxy)phenyl]-1H-pyrrole-2,5-dione; **13**, ergosterol.

The full profile of nucleosides and nucleobases was initially identified by matching the retention times and UV absorption profiles with respect to standards and was confirmed using ESI-MS. In total, ten compounds were identified in the water extract of mycelia AC. However, adenine, cytosine, and cytidine were not found when assessed using this new chromatographic condition, likely because their solubilities in the aqueous component of the mobile phase render poor column retention. Due to the bulky structures of these compounds (Figure 1), a specific extraction solvent and mobile phase were required for their coextraction and elution along with other compounds in the fingerprint. Our repeated trials for an optimal extraction showed that 100% methanol is the only choice capable of coextraction and elution. A gradient with 100% methanol was therefore adopted. In this way, different chemical compounds of various polarities are presented within the same chromatographic window despite the total elution time of all compounds lasting 120 minutes. Figure 3 shows the chromatographic fingerprint of methanol extract of mycelial AC.

### Preliminary application of mycelial AC chromatographic fingerprint

**Figure 4:**
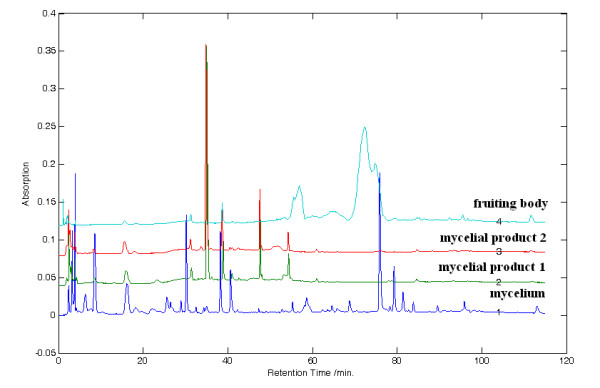
**The superimposed HPLC-DAD chromatograms of methanol extracts of two commercial mycelial products: crude mycelium and crude fruiting body of *Antrodia camphorata***. For the sake of clarity, numbering of compounds is not shown.

Two over-the-counter products that claimed consisted of mycelial AC were purchased from the Taiwanese market for our preliminary quality assessment. Figure 4 shows the superimposed chromatograms of methanol extract of the two commercial products in comparison to our reference fingerprint. The two commercial mycelial products possess very similar fingerprints, but these fingerprints are distinctively different from our established reference fingerprint of mycelial AC. From our morphological observation and confirmed by microscopic authentication during the species authentication stage, the powder in capsules are likely dried extracts rather than crude herbal material. The presence of additional possible herbal components other than those declared in the product package may also explain the difference in their derived fingerprints.

Moreover, the chemical compositions of mycelia and fruiting bodies have never been compared. The use of our chromatographic fingerprinting technique allowed a comparison of their chemical constituents. The fingerprint of the fruiting body part is distinctively different from that of the mycelium (Figure 4), suggesting there are different characteristic chemicals. In literatures, it suggested that the fruiting body is mainly composed of triterpenoids [29]. Therefore, specific reference chromatographic fingerprints should be used for independent quality control of the fruiting part of AC.

## Conclusions

This study provides the first chromatographic fingerprint to assess the quality of mycelial AC.

## Competing interests

The authors declare that they have no competing interests.

## Authors' contributions

Both authors took part in writing this manuscript. SSZ did the literatures review and all the experimental works. KSYL supervised on the project, advised and revised the manuscript. All authors read and approved the final version of the manuscript.
